# DNA Barcoding versus Morphological Variability of *Pterostichus brevicornis brevicornis* (Kirby, 1837) (Coleoptera, Carabidae) in the Arctic and Subarctic

**DOI:** 10.3390/insects13020204

**Published:** 2022-02-16

**Authors:** Natalia Andreevna Zubrii, Boris Yurevich Filippov, Alexander Vasilevich Kondakov, Olga Arturovna Khruleva, Leonid Borisovich Rybalov, Darya Vitalievna Vikhreva

**Affiliations:** 1 Scientific Department, Northern Arctic Federal University, Northern Dvina Emb. 17, 163002 Arkhangelsk, Russia; akondakv@yandex.ru; 2 Institute of Biogeography and Genetic Resources, N. Laverov Federal Center for Integrated Arctic Research of the Ural Branch of the Russian Academy of Sciences, Northern Dvina Emb. 23, 163000 Arkhangelsk, Russia; fby@yandex.ru (B.Y.F.); dvihreva@gmail.com (D.V.V.); 3 A.N. Severtsov Institute of Ecology and Evolution, Russian Academy of Sciences, Leninsky Prospekt, 33, 119071 Moscow, Russia; oa-khruleva@mail.ru (O.A.K.); lrybalov52@mail.ru (L.B.R.); 4 “Wrangel Island” State Nature Reserve, Kuvaeva, 23, 689400 Pevek, Chukotka Autonomous Region, Russia

**Keywords:** *Cryobius*, circumpolar range, phylogeny, phylogeography, genetic diversity, geometric morphometric

## Abstract

**Simple Summary:**

Taxonomic studies on a polymorphic species inhabiting a region with relatively uniform environmental conditions (e.g., the Arctic) should involve an integrative approach. Ground beetles such as the subgenus *Cryobius* of the genus *Pterostichus* are a successful group in expansion in the tundra biome. The current taxonomy of *Cryobius* species is unclear and could be considered an obstacle to ecological studies; knowledge of their distribution patterns in the Arctic is rather limited. In this study, the first report on the phylogeography and phylogeny of the most abundant tundra subspecies, *P.* (*Cryobius*) *brevicornis brevicornis* (Kirby, 1837), within its continuous range throughout northern Eurasia and North America is presented. The results indicated that the male genitalia morphology of *P. b. brevicornis* from Eurasian populations shared a higher geographic variability compared with the pronotum shape and the mitochondrial DNA sequences.

**Abstract:**

The geographic patterns of genetic and morphological variability in ground beetles were examined throughout Northern Eurasia and North America using the most abundant circumpolar tundra subspecies, *Pterostichus (Cryobius) brevicornis brevicornis* (Kirby, 1837), as a model. Phylogenetic structure was assessed on the basis of a Bayesian approach using two DNA markers (partial sequences of the COI and 28S rRNA genes), while phylogeographic patterns and population genetic diversity were estimated using the COI gene only. Morphological patterns were analysed using elliptical Fourier coefficients that were calculated based on the pronotum and male genitalia shape outlines. The subspecies shares 23 COI haplotypes throughout its entire circumpolar range, while eight haplotypes of 28S rRNA were detected in Northern Eurasia. Phylogenetic analysis did not reveal subdivided species lineages with strict geographical imprint. The network, F*_ST_* and uncorrected pairwise divergence analyses showed that the genetic distances between populations increase by longitude from Northeastern Asia to Europe. The genetic variability among the five studied geographical population groups of *P. b. brevicornis* was relatively high. The MANOVA showed significant regional divergence between local populations in Northern Eurasia based on both morphological markers, but only male genitalia variability was geographically structured. Neither the pronotum shape nor the male genitalia shape aligned with the phylogeographic patterns discovered on the basis of COI sequences. The genetic (COI) marker had more variation within, rather than among, population groups in addition to morphology of pronotum but not male genitalia.

## 1. Introduction

In the Arctic there are approximately 44 species of the genus *Pterostichus* (Bonelli, 1810) of which 20 species (45%) refer to the subgenus *Cryobius* (Chaudoir, 1838) [1,2,3]. The subgenus *Cryobius* is more diverse in the Palearctic region with 154 species, and 23 species have been detected in the Nearctic region [4,5,6]. In the last updated review of Carabidae, approximately 56 *Cryobius* species were detected in the Russian territory [7], with 15 *Cryobius* species endemic to this zone or predominantly distributed in tundra landscapes [3]. Danks also reported approximately 19 species of this subgenus in the American Arctic [8].

One of the first revisions of the subgenus *Cryobius* was published in 1906 by Poppius [9]. Since then, no more than 20 taxonomic research papers of the subgenera (species and supraspecies taxonomy) have been published. Three species groups within the subgenus *Cryobius* were determined for North America: *ventricosus*, *pinguedineus*, and *brevicornis* [10]. According to a study of ground beetles in the subgenus *Cryobius* in Russia and adjacent countries, five species groups and 17 species were defined outside such groups [11]. The last revision of the “brevicornis” group included five species: *Pterostichus brevicornis* (Kirby, 1837), *P. empetricola* (Dejean, 1828), *P. nivalis* (R.F. Sahlberg, 1844), *P. kolymensis* Erjiomin, 1998, and *P. mandibularoides* Ball, 1966 [12]. Additionally, the species *Pterostichus brevicornis* is divided into three subspecies: *Pterostichus brevicornis brevicornis* (Kirby, 1837), *Pterostichus brevicornis delicatus* (Casey, 1918), and *Pterostichus brevicornis yasudai* (Morita, 2002) [1]. The primary differences in morphology used to differentiate between species have focused on two body structures: the pronotum and male genitalia [10,12]. The general taxonomic problem of the subgenus *Cryobius* is the similar physiognomy of diagnostic features for species in each group as well as in the “brevicornis” group. Thus, limitations in descriptive taxonomy have resulted in biased estimates of ground beetle species compositions in the Arctic where the subgenus *Cryobius* is predominantly distributed. A contemporary integrative approach is required for clarification of these problems in taxonomy. Some research has been facilitated by molecular tools such as 28S rRNA, COI, and COII sequencing for the taxonomy of the subgenus *Hypherpes* (Chaudoir, 1838) of the genus *Pterostichus*, the widespread Pterostichini of North America [13]. Research using genetic data of *Cryobius* has focused on DNA barcoding of an arthropod community for environmental monitoring in the Arctic [14,15,16,17,18], on the molecular phylogeny of closely related taxa [13,19] in the study of the evolutionary trends of the genus *Pterostichus* [20], and on developing a comprehensive DNA barcode library of the genus *Pterostichus* for Germany and Central Europe [21].

*Pterostichus b. brevicornis* is one of the most widespread and numerous subspecies of the subgenus *Cryobius* in the northern polar region, including the territory from the Arctic tundra to the northern taiga subzones [7,10,11]. The subspecies’ range is almost circumpolar from Newfoundland to the Kola Peninsula and reaches the islands of the Arctic Ocean (Novaya Zemlya and Vaygach in Northern Europe, Wrangel in northeast Asia, Victoria in Canada) [10,11]. Southward, the species reaches the Amursko–Sakhalinskaya Mountainous Land in the east and the Buryatia Mountains in Siberia [11,22]. *Pterostichus b. delicatus* has a range restricted to the islands of the Bering Sea of North America and Northeast Russia [1,10,11]. Specimens of *Pterostichus b. yasudai* were detected in the Daisetsu Mountains of Hokkaido Island (Japan) [23]. *Pterostichus brevicornis* is a cold-adapted species [24,25] with a two-year life cycle, and has a “summer breeding” period and winter hibernation of imago and larvae [26]. Specimens of *P. b. brevicornis* from different parts of the subspecies range have a high morphological diversity of diagnostic features [10]. It might be assumed that in the Holarctic range, specific environmental adaptations and morphological diversity of *P. b. brevicornis* are caused by diversity at the genetic level. Therefore, the main goal of this study was to assess the genetic and morphological structures of *Pterostichus b. brevicornis* from Northern Eurasia and North America. The objectives were: (i) to study phylogenetic and phylogeographic aspects of *P. b. brevicornis*; (ii) to study the genetic diversity of *P. b. brevicornis* populations from different parts of the subspecies’ range; and (iii) to test the morphological differentiation of *P. b. brevicornis* within the Eurasian range with correspondence between morphological and genetic population structures.

## 2. Materials and Methods

### 2.1. Data Collection, DNA Extraction, PCR, and Sequencing

**Figure 1:**
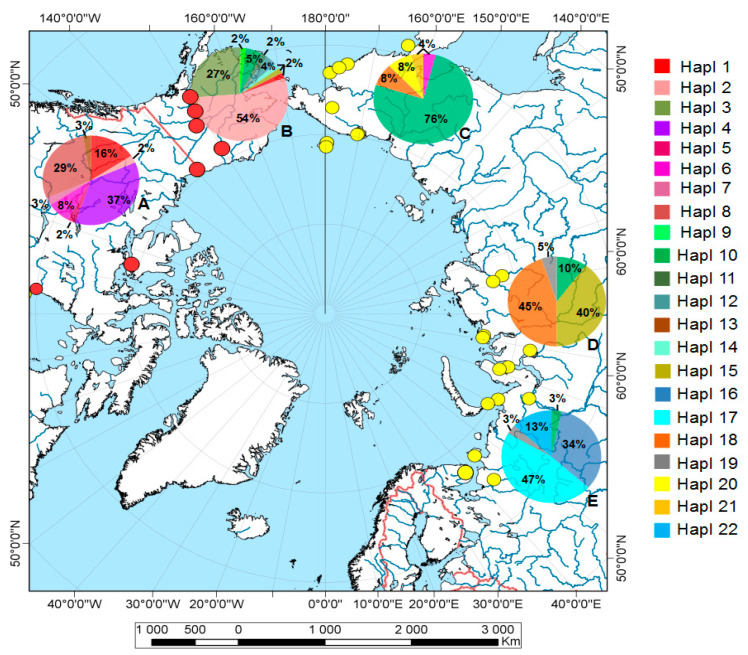
Map showing the location (dots) of *Pterostichus brevicornis brevicornis* samples (for details see Supplementary Materials Tables S1 and S3) and the respective mtDNA haplotype distribution (pie charts) per population: A—North Canada, B—Alaska, C—Northeast Asia, D—West and Central Siberia, E—North Europe. A unique COI haplotype from Kamchatka (Hapl 23) is not shown. Yellow dots—data from this study (genetic data and specimens for shape analysis); red dots—DNA sequences from NCBI GenBank.

Specimens of *P. b. brevicornis* were collected in various locations across North Eurasia and North America using pitfall traps, leaf litter sifting, and an exhaustor (Figure 1).

Carabid individuals for DNA analyses were preserved in 96% ethanol or dried after collection. The pinned dry specimens were deposited in the collection of the Russian Museum of Biodiversity Hotspots (RMBH), Federal Center for Integrated Arctic Research of the Russian Academy of Sciences and Northern (Arctic) Federal University named after M.V. Lomonosov, Arkhangelsk, Russian Federation. In this study, the cytochrome *c* oxidase subunit I (COI) gene and large subunit ribosomal RNA 28S (28S rRNA) gene sequences were obtained. The newly obtained sequences of *P. b. brevicornis* (77 for COI and 33 for28S rRNA) were combined with published GenBank data (93 for COI). The resulting dataset comprised a total of 170 sequences for COI and 33 sequences for 28S rRNA (Supplementary Materials Table S1).

DNA was extracted from legs and dissected abdominal muscle tissue using a standard phenol/chloroform procedure [27]. A fragment of the mitochondrial gene COI was amplified using two primer pairs: C1-J-1718 with C1-N-2329 (612 bp) [28] and LCO 1490 [29] with LepR (660 bp) [30]. The primers D23F [31] and D2 [32] were used for the amplification of the 28S rRNA gene (528 bp). The PCR mix contained approximately 200 ng of total DNA, 10 pmol of each primer, 200 μmol of each dNTP, 2.5 μL of PCR buffer (with 10 × 2 mmol MgCl_2_), 0.8 units of Taq DNA polymerase (SibEnzyme Ltd., Novosibirsk, Russia), and H_2_O added for a final volume of 25 μL. Thermal cycling was as follows: 95 °C (4 min), 32–37 cycles of 95 °C (50 s) and 42 °C (50 s) for COI or 25 cycles of 95 °C (50 s), 60 °C (50 s) for 28S, 72 °C (50 s), and a final extension at 72 °C (5 min). Forward and reverse sequencing were run on an ABI PRISM^®^ 3730 DNA (Thermo Fisher Scientific Inc., Waltham, MA, USA) using the ABI PRISM^®^ Big-Dye Terminator v. 3.1 reagent kit. Regarding the results, 85 new sequences (61 for COI and 24 for 28S rRNA) were deposited in GenBank (accession numbers in Table S1). In addition, COI sequences of *P. b. brevicornis* were retrieved from GenBank (NCBI) and the Barcode of Life Data (BOLD) System. All sequences were checked manually using BioEdit v. 7.2.5 [33]. The alignment of the COI and 28S rRNA sequences was performed using the Muscle algorithm in MEGA X [34]. For the phylogenetic analyses, COI and 28S rRNA sequence alignments were trimmed, leaving 597 bp and 524 bp fragments, respectively.

### 2.2. Phylogenetic and Phylogeographic Analyses

Both genes were analysed separately (Supplementary Materials Table S2, Figure S1) and combined. The aligned sequences of COI and 28S rRNA were concatenated in a single nucleotide sequence alignment (total length of 1121 bp). This combined dataset was collapsed into 28 unique haplotypes (including two of the outgroup taxa—*Pterostichus nivalis* (R.F. Sahlberg, 1844)) using an online FASTA sequence toolbox [35]. For 28S rRNA alignment, the online GBlocks server v0.9b [36] was applied to eliminate poorly aligned positions and divergent regions from the alignment. The best models of sequence evolution for each partition, as suggested based on the corrected Akaike Information Criterion (AICc) [37] of MEGA X [34], were as follows: (1) 1st codon position of COI: TN93+G (G = 0.05), (2) 2nd codon position of COI: TN93, (3) 3rd codon position of COI: GTR+G (G = 0.2), and (4) 28S rRNA:TN93+G+I (G = 0.05, I = 0.59). Phylogenetic relationships were reconstructed based on Bayesian inference performed in MrBayes v. 3.2.6 [38] through the CIPRES Science Gateway [39]. The following parameters for analyses were used: ngen = 10,000,000, nchains = 4, nruns = 4, samplefreq = 1000, temp = 0.2. The first 15% of trees were discarded as burn-in (preconvergence part), and the majority rule consensus tree was calculated from the remaining trees. Convergence of the MCMC chains to a stationary distribution was checked visually based on the plotted posterior estimates using Tracer v. 1.7 [40]. The effective sample size (ESS) value for each parameter sampled from the MCMC analysis was recorded as >700. Trees were viewed using FigTree v. 1.4.2 [41].

The phylogeographic analysis was performed based on a median-joining network approach using Network v. 5.0.0.1 software with default settings [42]. Additionally, 170 COI sequences of *P. b. brevicornis* from 26 localities of Northern Europe and North America were used (Supplementary Materials Table S1). All sequences were trimmed to the minimal sequence length (602 bp) due to different length of available sequences. Additionally, pie charts of haplotype contributions (in percent of total abundance) per geographical population were added to the map of sample locations of *P. b. brevicornis* (Figure 1) created with QGIS software version 3.14.1 (https://qgis.org/ru/site/, accessed on 27 July 2021).

### 2.3. Population Genetic Analysis

Population genetic diversity indices (haplotype and nucleotide diversity) were determined using Arlequin v.3.5.2 [43]. The total dataset of 168 COI sequences was subdivided in accordance with geographic areas of North Canada: Nunavut, Manitoba, Yukon (*n* = 38), Alaska: Toolik, Talkeetna, Fairbanks Borough, Denali Borough (*n* = 55), Northeast Asia: Wrangel Island and Chukotka (*n* = 25), West and Central Siberia: Yamal Peninsula and Polar Ural, Tazovskiy and Taymir Peninsulas (*n* = 20), Northern Europe: Pinega Reserve, Kanin Peninsula, Yugorskiy Peninsula, Vaigach Island (*n* = 30). Pairwise F*_ST_* values using analysis of molecular variance (AMOVA) for comparisons of five population groups were calculated using Arlequin v.3.5.2. The mean uncorrected p-distance between population groups (±standard error estimate) was calculated for COI using MEGA X.

### 2.4. Morphological Studies

The morphological study for *P. b. brevicornis* mainly follows a redescription of Ball [10] and Erjiomin [12]. The comparative analysis of the morphology variabilities in the longitudinal range of North Eurasia of our collected materials was conducted with attention to the shape of pronotum and male genitalia (aedeagus) (Table S3). Both morphological structures were analysed using elliptical Fourier coefficients (EFs) [44,45,46,47,48,49], which were calculated using the software package SHAPE v.1.3 [50]. The EF geometric morphometric (GM) approach is advantageous over the traditional method of measurement (linear distances between predetermined points) and can compare curvilinear features with object size inclusion [49,50]. The pronotum shape analysis was performed for a total of 116 individuals (49 males and 67 females) from 17 sampling sites (Table S3). Male genitalia shape was investigated in 49 individuals from 10 populations (Table S3). The images of the morphological details were taken with a stereomicroscope (AXIO Zoom V16, Carl Zeiss, Germany) and digitized by ZEN 2.3 (blue edition) software [51]. The resulting images were edited in Adobe Photoshop CC 2014 [52] to produce black and white bitmaps. Elliptic Fourier descriptors (EFDs) based on the longest radius were calculated using the Chc2Nef (number of harmonics—20) program [50]. A principal components analysis (PCA) was implemented on the variance-covariance matrix from the EFD coefficients using the PrinComp program [50]. The shape variations of the pronotum and aedeagus were explained by principal component (PC) scores (±2 standard deviations). All significant PC scores were used in subsequent analyses.

Correspondence between principal component scores (shape variables) as dependent variables due to species populations, sex, or population × sex interaction was investigated using multivariate analysis of variance (MANOVA). MANOVA was also used to test the mean difference in shape markers between geographical populations. Discriminant canonical analysis (DCA) was also performed with populations as the dependent variable. The purpose was to illustrate the maximum differentiation of the multivariate means (centroids) of morphological patterns for each local population sample in shape space. The matrices of principal component scores for both morphological structures were projected onto computed canonical variate axes. Data analysis was conducted using both STATISTICA v. 10 [53] and PAST v. 3.06 [54].

The relationship between morphological, genetic, and geographical differentiation was assessed by testing simple and partial Mantel tests between distance matrices. Morphological distances (shape distance) corresponding to Mahalanobis distances (D_M_) by DCA were calculated in STATISTICA v. 10 [53]. Genetic distances were based on mean pairwise population F*_ST_* values using the Tamura model for the barcoding segment of COI and were calculated in Arlequin v. 3.5.2 [43]. The geographical distance matrix was determined using the great circle distance between collected samples in PAST v. 3.06. The only samples with both genetic and shape datasets (16 for pronotum and 10 for male genitalia) were involved in the Mantel tests of matrix correlation (Supplementary Materials Table S2). All tests in PAST v. 3.06 were based on 10,000 permutations of the data to test the null hypothesis that the matrices are independent.

## 3. Results

### 3.1. Phylogenetic Reconstruction, Phylogeography, and Population Genetics

**Figure 2:**
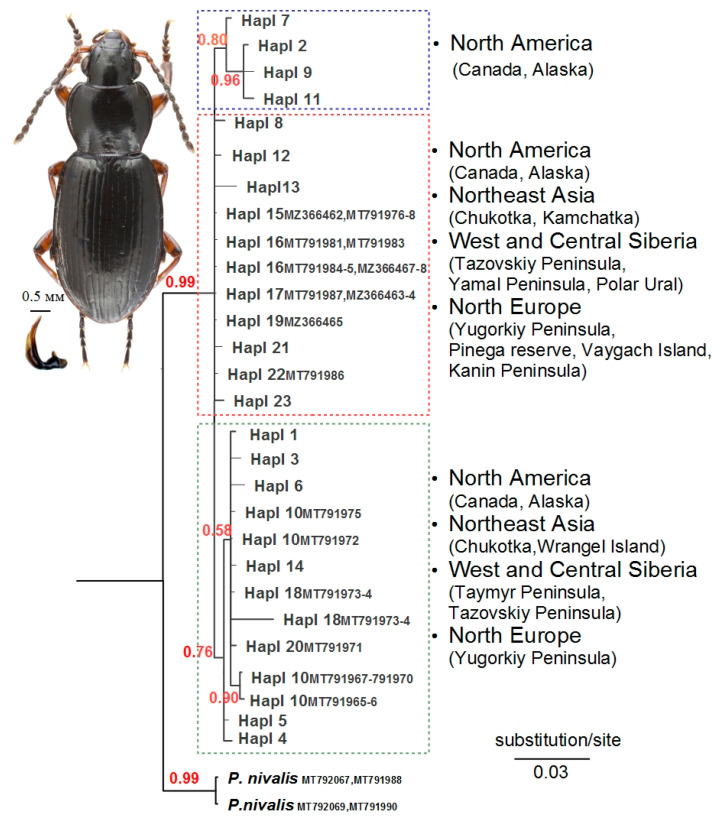
The majority-rule consensus phylogenetic tree of *Pterostichus brevicornis brevicornis* recovered from Bayesian inference analysis based on the combined mitochondrial and nuclear sequence dataset. Haplotype codes are indicated in Supplementary Materials Table S1 (on tree plot haplotype numbers according to COI marker). Numbers near branches indicate the Bayesian posterior probability (BPP). Photo by Zubrii N.A (male specimen; Russia, Yugorskiy Peninsula, near Amderma settlement).

The Bayesian phylogeny of *P. b. brevicornis* based on 23 COI and eight 28S rRNA unique haplotypes comprised three subclades that were 0.7–0.9% divergent (Figure 2).

There were no intraspecific subclades of *P. b. brevicornis* by longitude range distribution. Only one subclade combined four haplotypes from North America (Alaska and Canada (Yukon)), while others contained haplotypes from different geographical localities. The COI mean uncorrected pairwise p-distance between five geographical populations as a whole was 0.44 ± 0.17%. Genetic distances increased from east to the west in Eurasia, and the highest mean divergence value (p-distance: 1.24 ± 0.41%; F*_ST_* = 0.83, *p* < 0.000) was found between Northern European and Northeastern populations (Table 1). However, sequence variation of 28S rRNA of Eurasian populations differed from mtDNA, with the highest mean divergence value between Northeastern and both north European and Siberian populations (p-distances: 1.20% to 1.30%), and the lowest between north European and Siberian populations (p-distances: 0.16 ± 0.12%). The mean 28S rRNA p-distance between the three Eurasian populations as a whole was 0.79 ± 0.19%.

**Figure 3:**
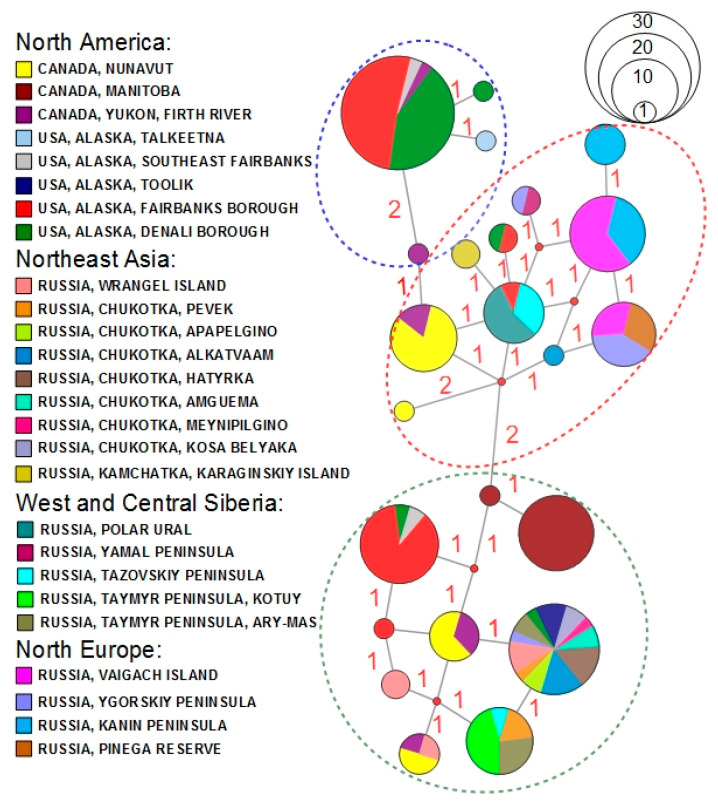
Phylogeography of *Pterostichus brevicornis brevicornis.* Median-joining network of COI sequences (see Supplementary Materials Table S1 for details). Colours match locations in the legend and mutations are shown as red numbers at the branches. The dashed ellipses correspond to subclades of the phylogenetic tree (see Figure 2). The relationship between circle size and number of specimens is shown in the upper right corner.

The COI sequences of *P. b. brevicornis* from Europe and Northeast Asia had more genetic similarities with samples from North America than with each other (Table 1). It is noteworthy that the Canadian population of *P. b. brevicornis* had lower genetic distances with subspecies population from West and Central Siberia (p-distance: 0.72 ± 0.23%; F*_ST_* = 0.19, *p* < 0.000) than with those from Alaska (p-distance: 0.82 ± 0.26%; F*_ST_* = 0.28, *p* < 0.000). The median-joining network analysis using the short COI sequence dataset corresponded to the Bayesian phylogeny with closely related haplotypes among *P. b. brevicornis* from different localities of the northern range (Figure 3).

Overall, 16 COI haplotypes of *P. b. brevicornis* were found in North America, with 12 unique haplotypes (75% of the total), and 11 COI haplotypes were determined for Eurasia, with eight unique haplotypes (72.7%). Only three COI haplotypes were shared between Nearctic and Palearctic populations of *P. b. brevicornis*: Hapl 6 in Canada and the Northeast Asia, Hapl 10 in all studied territories except Canada, and Hapl 15 in Alaska and Siberia. The most widespread haplotype (Hapl 10) was found in approximately half of the studied locations (12 locations) within North America and Eurasia (Figure 1 and Figure 3). Unique haplotypes were found with high frequency in the most extreme extents of the eastern and western territories of the northern longitudinal range of *P. b. brevicornis* in North Canada (Hapl 4, 36.8%) and in northern Europe (Hapl 16, 33.3%; Hapl 17, 46.7%) (Figure 1). The highest divergence of up to 10–11 mutational steps was detected within the Alaska populations (Figure 3). In comparison, the subdivided Eurasian populations had lower divergence (maximum seven steps) for each of the subgroups (Figure 3). Tests of genetic structure using AMOVA were significant (F*_ST_* = 0.48, df1 = 4, df2 = 163, *p* < 0.000), with the highest variation found within population groups (51.99%) compared to the variation among population groups (48.01%).

For the North Canadian population of *P. b. brevicornis,* the highest haplotype diversity (h > 0.7) was detected, yet there was also low nucleotide diversity (π < 0.6%). The studied samples of *P. b. brevicornis* from Siberia and Alaska were characterized by lower haplotype diversity (h < 0.7) and the highest nucleotide diversity (π > 0.6%). Lower values of both population parameters (h < 0.7, π < 0.5%) were detected for *P. b. brevicornis* from Northern Europe and Northeast Asia (Table 2).

### 3.2. Shape Variation

The total shape variation for both the pronotum and aedeagus of *P. b. brevicornis* comprised 38 principal components, but only five were significant. The first five PCs described 90.22% of the shape variation in the pronotum and 87.98% of the variation in male genitalia (Supplementary Materials Table S4).

The MANOVA indicated no significant difference in the shape of pronotum male and female species individuals or in the shape of pronotum between sexes by population (Table 3).

Highly significant differences in pronotum and aedeagus shapes of *P. b. brevicornis* were found between local populations (Table 3) by all principal component axes. The centroids of the PCs of both morphological structures for the studied populations were calculated using DCA. Five statistically significant canonical axes were established, and the first two of them explained 74.8% of the variance for pronotum shape and 78.6% of the variance for aedeagus shape (Table 4). 

**Figure 4:**
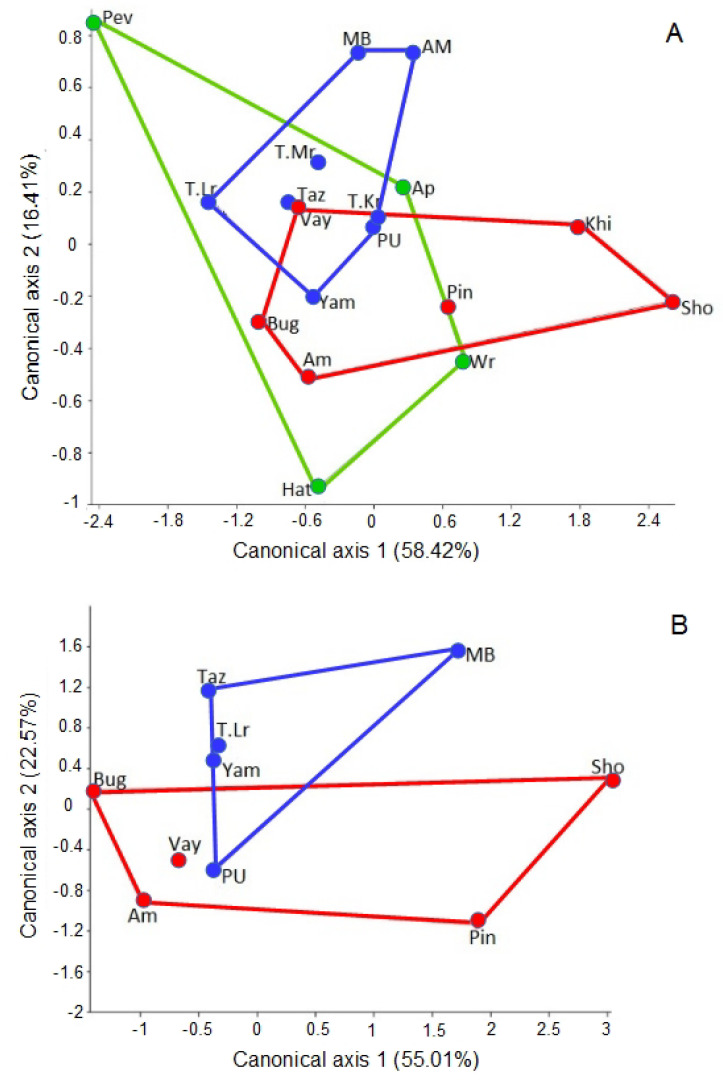
Plot of the population centroids onto the first two canonical axes for the pronotum (**A**) and the aedeagus (**B**) shape of *Pterostichus brevicornis brevicornis* (for population abbreviations see Supplementary Materials Table S3). Spot and polygon colours: red—North Europe; blue—West and Central Siberia; green—Northeast Asia.

The scatterplot of population mean scores for pronotum shape on the first two canonical axes showed three overlapping groups of populations (Figure 4A).

**Figure 5:**
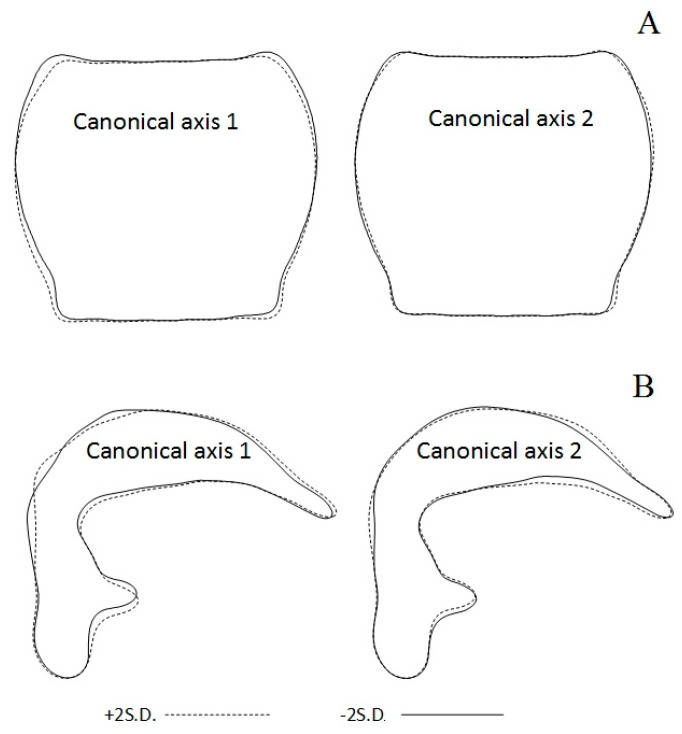
Pronotum (**A**) and aedeagus (**B**) shape variations of *Pterostichus brevicornis brevicornis*. Outlines were reconstructed, and the principal component scores with standard deviation values (±2 SD) were represented for the first two canonical axes (for details see Table 4).

The nine local populations from Northern Europe, West and Central Siberia, and Northeast Asia belong to the overall shape space (Figure 4A). The three groups of subdivided geographical populations of *P. b. brevicornis* had more variation in pronotum shape within than among groups for all canonical axes (Wilk’s Lambda = 0.538, F = 0.798, df1 = 10, df2 = 22, *p* = 0.632). Specimens from the Taymyr Peninsula (Maksimovka River) and Vaygatch Island had similar population mean scores for pronotum PCs. The greatest distances between PC centroids of pronotum shape were detected on the first axis for populations from Chukotka (Pevek settlement) and Kanin Peninsula (Shoina settlement) (Figure 4A). Reconstructed outlines of pronotum on the first axis showed side rounded, almost to hind angle shapes for positive projections, sides that were not strongly rounded, and feebly sinuate posterior shape for negative projections (Figure 5A).

For the second axis shape, changes of pronotum were not so obvious and slight asymmetry of sides and hind angle shapes for negative projections were observed (Figure 5A).

For the aedeagus shape of *P. b. brevicornis*, the MANOVA including all five canonical components (mean scores for PCs) revealed more variance within than among population groups of Northern Europe and Siberia (Wilk’s Lambda = 0.517, F = 0.749, df1 = 5, df2 = 4, *p* = 0.628). A significant difference in aedeagus shape among the population groups of the studied territories resulted from a second canonical axis only (Mann–Whitney: U = 3.00, *p* = 0.042, *n* = 10). The population mean scores of aedeagus shape on the scatterplot for the second canonical axis were not completely separated in shape space between Northern Europe and Siberia (Figure 4B). The aedeagus shapes of *P. b. brevicornis* from the Polar Ural sample had more similarity with shapes of aedeagus samples from Vaygach Island than with male genitalia samples from West and Central Siberia (Figure 4B). Reconstructed aedeagus outlines of the left lateral aspect for the first axis showed that the aedeagus shape for positive projections had a sharper bend for the median lobe and a more sloping obtuse-angled bend near the base of the median lobe for negative projections (Figure 5B). For the second axis, there was a thicker apical portion of the median lobe for positive projections but a thinner apical portion of the median lobe for negative projections (Figure 5B). On the scatterplot, the positions in the negative projections of the second axis gathered Northern European populations, and the samples from West and Central Siberia were predominantly placed in the positive projections (Figure 4B).

The simple Mantel test supported a correlation between the genetic and geographical distances (r = 0.301, *p* = 0.013, *n* = 16). The results of correlations between distance matrices were different for the pronotum and aedeagus morphology of *P. b. brevicornis* (Table 5).

The pronotum shape-distance matrix was not significantly correlated with either geographical or genetic distances. However, the correlation between geographical and genetic distance when correcting for pronotum morphology was significant. For male genitalia, the morphological and geographical distances were significantly correlated (r = 0.600, *p* = 0.033, *n* = 10). However, there was no significant correlation between morphological and genetic distance. The partial Mantel tests showed the same pattern where genetic differentiation did not correspond with aedeagus morphology (Table 5). Therefore, the male genitalia morphology of *P. b. brevicornis* among Eurasian populations was more structured than the pronotum shape and mtDNA.

## 4. Discussion

For *P. b. brevicornis*, 23 unique COI haplotypes throughout the circumpolar range and eight unique haplotypes of 28S rRNA in North Eurasia were detected (Supplementary Materials Table S1). A higher number of COI haplotypes were detected in North American samples (16 haplotypes) than in the Eurasian populations (11 haplotypes). Among the studied COI dataset, a high proportion of unique haplotypes (up to 75%) and only three common haplotypes were revealed for both continents. The value of mtDNA uncorrected pairwise divergence within a subspecies across the circumpolar range was approximately 0.5%, and pairwise divergence 28S rRNA was approximately 0.8% for the North Eurasian part of the range. Although *P. b. brevicornis* had a broad range in the Polar region with insular populations, the analysis of genetic structure did not reveal divided subspecies lineages with strict geographical patterns. The phylogenetic tree based on concatenated COI and 28S rRNA sequences and the network of mtDNA showed subclades and haplotypes that combined sequences from different locations within the Polar region. Despite subdivided population groups of *P. b. brevicornis* having a high percentage of unique COI haplotypes, both the tests of genetic structure and phylogeography analysis detected higher genetic similarities among rather than within groups (11 mutation steps between haplotypes of the mountain population of southern Alaska). However, the mtDNA network, F*_ST_* and uncorrected pairwise divergence analysis revealed an increase in genetic distances by longitude from Northeast Asia to North European populations. The haplotype network configuration had new haplotypes that were derived from low divergent haplotypes by one or two mutation steps that indicated a common population history with relatively recent mutation events [55]. The COI haplotype (Hapl 10, 14.7% of the studied sequences) with the highest frequency in tundra populations of Chukotka and Wrangel Island was shared with Northern Europe (Yugorsky Peninsula), Siberia (Tazovsky Peninsula and Taymyr Peninsula) and Alaska (Denali Borough, Toolik). The insular populations of *P. b. brevicornis* of the Arctic islands shared COI haplotypes with adjacent mainland populations (Wrangel and Chukotka populations, Vaygach and Yugorsky Peninsula populations). Similar results were detected for other insect taxa with continuous ranges in the Arctic, such as the tiger moth *Arctia tundrana* (Tshistjakov, 1990), which has low genetic divergence among populations from Kolguev Island to Chukotka [56]. The bumblebee *Bombus pyrrhopygus* Friese, 1902 from Novaya Zemlya, had a single COI haplotype with populations from Norway and Kamchatka [57]. The Arctic bumblebee *Bombus glacialis* Friese, 1902 from Wrangel shared three unique COI haplotypes and was closely related to those inferred from samples from Novaya Zemlya [58]. The observed patterns indicated complicated processes of colonization of insect faunas in the Arctic after the Last Glacial Period (LGP) with postglacial invasions from dispersed southward populations and from montane refugia [59].

Analysis of mtDNA haplotypes showed overall high genetic variability within the studied geographical populations of *P. b. brevicornis*: four to nine haplotypes and 0.42–0.77 gene diversity. Lower genetic diversity was detected for the Northeastern (h < 0.5, π < 0.3%) and European populations (h < 0.7, π < 0.3%) of *P. b. brevicornis*. Higher genetic diversity was revealed for populations from West and Central Siberia, Alaska (h > 0.6, π > 0.6% for both), and North Canada (h > 0.7, π < 0.6%), which were likely associated with the ecological plasticity of these populations of *P. b. brevicornis* [60]. 

The pronotum and male genitalia morphologies of Northern Eurasian populations of *P. b. brevicornis* did not correspond with patterns of mtDNA variation. Both morphological structures had significant shape differences between local populations in Northern Eurasia, but only significant divergences were detected for the aedeagus shape between subdivided geographical populations of Northern Europe and Siberia. Correspondence between morphological distance and geographical distance was significant for the aedeagus but not associated for the pronotum. Genitalia are the fastest evolving characters in insects and have phylogenetic implications for *Pterostichus* species [20]. For *Ohomopterus* ground beetles, mechanical agents of reproductive isolation (body size and genital morphology) provide fast species radiation without substantial ecological differentiation on the Japanese Islands [61]. Additionally, research into the evolutionary history of genital diversification and speciation by reconstructing phylogenetic relationships among three pairs of parapatric species of *Carabus* (*Ohomopterus*) beetles demonstrated that species diversification can follow the coevolution of genitalia between the sexes [62]. In this case, the geographical structure of genitalia morphology would be established before the genetic structure of a rapidly evolving neutral marker [46]. For *P. b. brevicornis*, the geographic structure of male genitalia can indicate such a pattern. However, to test this hypothesis, data samples from larger numbers of populations by circumpolar range were required. For both genetic markers, sequence divergences between North European and Siberian populations were not above any of the thresholds. Sexual dimorphism of the pronotum shape was not found for North Eurasian populations. According to Ball [10], the pronotum shape of *P. b. brevicornis* was divided into four classes by measures of side rounding (from not strongly rounded to strongly rounded) and sinuate hind angles (from feebly sinuate to markedly sinuate). The studied samples revealed pronotum shapes from the 1st to 4th classes with a predominating position of the samples at the gradient centre. In a previous revision, it was mentioned that there were a majority of second and third classes of pronotum shape within Northern Eurasia for specimens from Chukotka (Pitlekai Cape, Uelen settlement (Whalen Island)), Central Siberia (Yenisey Basin), and Northern Europe (Vaygach Island and Novaya Zemlya) [10]. The result was close to previous conclusions of Ball about the pattern of distribution of pronotum shape of *P. b. brevicornis* in Eurasia, and the marked variability of this morphological marker was confirmed statistically. Therefore, aedeagus shape was a stronger morphological structure for *P. b. brevicornis* taxonomy, and pronotum shape can hardly be used as a marker for distinguishing *P. brevicornis* subspecies.

Previous research of one species or sister species populations of insects also confirmed that morphological markers were not more structured than genetic patterns and did not always correlate [44,46,63]. Analysis of sister mosquito species of the subgenus *Culex* revealed wing shape differences between populations, but genetic markers (COI, NADH5, CAD, Hunchback) did not corroborate the morphological variables [64]. A similar result was detected for sister species in the butterfly genus *Lycaeides* (Hübner, 1819), where strong habitat and male genitalia structure partitions were detected but not by using mtDNA data [65]. The pronotum and aedeagus shape variabilities of the ground beetle *Carabus solieri* (Deuve, 1994) among populations in the southern Alps of France and the Ligurian Alps of Italy were less structured than genetic data (mtDNA), but the markers were congruent [44]. In the scarab beetle *Phyllophaga hirticula* (Knoch, 1801) from the eastern USA, female genitalia were more geographically structured than mtDNA and male genitalia [46].

However, current research has shown the limitation of using DNA sequences for species identification if species pairs have very recent origins or hybridize [66]. Such patterns were shown for two pairs of closely related *Pterostichus* species: no significant sequence divergence within three nuclear markers and mtDNA for *P. nigrita* and *P. rhaeticus* were revealed [66], and similarly a lack of mitochondrial divergence between *P. oblongopunctatus* and northern populations of *P. adstrictus* was detected [67]. In this case, next-generation sequencing (NGS) methods were more informative [68], and the restriction site-associated DNA sequencing method (RAD-seq) was more informative [62,68,69,70,71]. However, for the fly genus *Chiastocheta* (Pokorny, 1889), RAD-seq-based phylogenies showed limitations for reconstructing interspecific relationships among recently diverged lineages (less than 1.6 Mya) [71]. For the Holarctic subspecies complex of *P. brevicornis* with a relatively recent history of populations in the Arctic after LGP within Northern Europe and Western Siberia [59,72], nuclear sequence data is probably less informative in explaining the observed patterns in morphology but may be more informative for Northeastern and North American subspecies populations. Ball suggested more subspecies of *P. brevicornis* within North America mainland but did not divide them due to high morphological variability within populations rather between them [10]. This again confirms the necessity of an integrative approach in insect taxonomy and geometric morphometric analyses as a useful tool in obtaining intraspecific phenotypic variability within taxa and especially for taxonomic, phylogenetic, or ecological issues [73].

## 5. Conclusions

*Pterostichus b. brevicornis* is a polymorphic subspecies with high genetic variability throughout the circumpolar range of North Eurasia and North America. The COI marker had more variation within, rather than among, population groups in addition to the morphology of the pronotum but not male genitalia.

## Figures and Tables

**Table 1:** Mean genetic divergences between population groups of *Pterostichus brevicornis brevicornis* (COI uncorrected p-distance ± standard error estimations, %) on the lower diagonal and F*_ST_* values on the upper diagonal (significance of all measures *p* < 0.0001).

Populations	North Europe	West and Central Siberia	Northeast of Asia	Alaska	North Canada
North Europe	0	0.59	0.83	0.55	0.49
West and Central Siberia	0.95 ± 0.31	0	0.27	0.35	0.19
Northeast of Asia	1.24 ± 0.41	0.57 ± 0.20	0	0.57	0.47
Alaska	1.01 ± 0.33	0.98 ± 0.30	1.09 ± 0.33	0	0.28
North Canada	0.76 ± 0.27	0.72 ± 0.23	0.73 ± 0.25	0.82 ± 0.26	0

**Table 2:** Genetic diversity indices of *Pterostichus brevicornis brevicornis* samples based on COI sequences.

Parameters	North Europe	West and Central Siberia	Northeast of Asia	Alaska	North Canada
Sample size, *N*	30	20	25	55	38
No. of haplotypes	5	4	5	9	8
Haplotype diversity (h ± SD)	0.674 ± 0.055	0.658 ± 0.070	0.423 ± 0.119	0.634 ± 0.055	0.767 ± 0.042
Nucleotide diversity (π ± SD), %	0.208 ± 0.15	0.640 ± 0.38	0.207 ± 0.15	0.638 ± 0.36	0.541 ± 0.32

**Table 3:** Multivariate analysis of variance (MANOVA) performed for shape variables of pronotum and aedeagus of *Pterostichus brevicornis brevicornis* (df1—model degrees of freedom; df2—error degrees of freedom). Differences statistically significant at *p* < 0.05.

Body Units	Effect	Wilks’s Lambda	df1, df2	F	*p*
Pronotum	Sex	0.916	5, 59	1.248	0.223
	Population	0.110	45, 267	2.272	0.0000
	Sex × Population	0.364	45, 267	1.502	0.056
Aedeagus	Population	0.109	45, 155	2.207	0.0002

**Table 4:** The structure matrix of shape variables (principal component scores—PC) of canonical discriminant analysis explained by canonical axes (CA).

Shape Variables	CA1	CA2	CA3	CA4	CA5
Pronotum					
PC1	0.026	**−0.701**	0.458	0.052	0.544
PC2	−0.337	−0.063	−0.058	−0.933	0.091
PC3	0.041	−0.019	−0.708	0.081	0.700
PC4	**−0.657**	−0.334	−0.244	0.557	−0.295
PC5	−0.275	0.597	0.432	0.198	0.584
Cumulative proportion	0.584	0.748	0.882	0.958	1.000
Aedeagus					
PC1	0.099	−0.011	−0.717	−0.674	−0.149
PC2	−0.087	**0.794**	−0.350	0.432	−0.231
PC3	−0.222	0.004	0.246	−0.277	−0.902
PC4	**0.763**	0.321	0.452	−0.214	−0.254
PC5	0.238	−0.414	−0.405	0.604	−0.493
Cumulative proportion	0.550	0.786	0.889	0.972	1.000

bold: maximum PC contribution.

**Table 5:** Summary of simple (r) and partial (r′) Mantel tests for correspondence between morphological distances (Shape) and genetic (Gen) or geographic distances (Geo).

	Pronotum		Aedeagus	
	r	*p*	r	*p*
Simple Mantel Tests				
Shape × Gen	0.089	0.287	0.199	0.288
Shape × Geo	0.218	0.147	0.600	0.033
Partial Mantel Tests				
Shape × Gen (Geo)	−0.147	0.789	0.332	0.091
Shape × Geo (Gen)	0.253	0.124	0.579	0.032
Geo × Gen (Shape)	0.347	0.006	0.332	0.093

## Data Availability

The data presented in this study are available in the article and Supplementary Materials.

## Supplementary Materials

The following are available online at https://www.mdpi.com/article/10.3390/insects13020204/s1 Table S1: List of sequenced specimens of *Pterostichus b. brevicornis* and *Pterostichus nivalis*, including the location wiht BOLD and NCBI’s GenBank accession numbers, Table S2: *Pterostichus brevicornis brevicornis* 28S rRNA polymorphism*, Table S3: Sampling sites and sample size: total number of pronotum (males and females) and male genitalia (aedeagus) of *Pterostichus b. brevicornis*, Table S4: Contribution of significant principal components to shape variation of pronotum and aedeagus of *Pterostichus b. brevicornis*, Figure S1: Phylogenetic tree of *Pterostichus brevicornis brevicornis* recovered from Bayesian inference analysis based on the COI sequence dataset. Haplotype codes are indicated in Table S1. The dashed rectangles corresponded to subclades of the majority-rule consensus phylogenetic tree (see Figure 2).Numbers near branches indicate the Bayesian posterior probability (BPP).
