# Supplementary material for: DNA Barcoding versus Morphological Variability of Pterostichus brevicornis brevicornis (Kirby, 1837) (Coleoptera, Carabidae) in the Arctic and Subarctic
Source: Insects. 2022 Feb 16;13(2):204. doi: 10.3390/insects13020204 (PMC8876867; doi:10.3390/insects13020204)
Supplement: Supplementary file 1 [file insects-13-00204-s001.zip › insects-1374564-supplementary.pdf]

insects MDPI

Supplementary Materials:

DNA Barcoding versus Morphological Variability of *Pterostichus brevicornis brevicornis* (Kirby, 1837) (Coleoptera, Carabidae) in the Arctic and Subarctic

Natalia Andreevna Zubrii, Boris Yurevich Filippov, Alexandr Vasilevich Kondakov, Olga Arturovna Khruleva, Leonid Borisovich Rybalov and Darya Vitalievna Vikhreva

Table S1. List of sequenced specimens of *Pterostichus b. brevicornis* and *Pterostichus nivalis*, including the location wiht BOLD and NCBI's GenBank accession numbers.

Species	COI Haplotype Code	GenBank / BOLD IDS acc. no. (COI)	GenBank / BOLD IDS acc. no. (28S rRNA)	Locality	Data Source*
Pterostichus brevicornis brevicornis (*Kirby,1837*)	Hapl1	MN673460.1 ACHAR803-18	n/a	Canada, Nunavut	Pentinsaari, 2020
P. b. brevicornis	Hapl1	MN670020.1 ACHAR781-18	n/a	Canada, Nunavut	Pentinsaari, 2020
P. b. brevicornis	Hapl1	MN679965.1 ACHAR828-18	n/a	Canada, Nunavut	Pentinsaari, 2020
P. b. brevicornis	Hapl1	HQ961899.1 JSYKA240-10	n/a	Canada, Yukon, Firth River	GenBank BOLD IDS
P. b. brevicornis	Hapl1	HQ961900.1 JSYKA241-10	n/a	Canada, Yukon, Firth River	GenBank BOLD IDS
P. b. brevicornis	Hapl1	MN672408.1 ACHAR877-18	n/a	Canada, Nunavut	Pentinsaari, 2020
P. b. brevicornis	Hapl6	ACHAR876-18	n/a	Canada, Nunavut	BOLD IDS
P. b. brevicornis	Hapl6	MN670684.1 ACHAR868-18	n/a	Canada, Nunavut	Pentinsaari, 2020
P. b. brevicornis	Hapl8	FCHAR1280	n/a	Canada, Nunavut	GenBank
P. b. brevicornis	Hapl8	FCHAR2131	n/a	Canada, Nunavut	BOLD IDS
P. b. brevicornis	Hapl8	FCHAR6620	n/a	Canada, Nunavut	GenBank
P. b. brevicornis	Hapl8	FCHAR7057	n/a	Canada, Nunavut	BOLD IDS
P. b. brevicornis	Hapl8	FCHAR7369	n/a	Canada, Nunavut	GenBank
P. b. brevicornis	Hapl13	FCHAR7721	n/a	Canada, Nunavut	BOLD IDS
P. b. brevicornis	Hapl8	FCHAR1303	n/a	Canada, Nunavut	GenBank
P. b. brevicornis	Hapl8	FCHAR1618	n/a	Canada, Nunavut	BOLD IDS
P. b. brevicornis	Hapl8	FCHAR2132	n/a	Canada, Nunavut	GenBank
P. b. brevicornis	Hapl8	FCHAR5467	n/a	Canada, Nunavut	BOLD IDS
P. b. brevicornis	Hapl2	JSYKA235-10	n/a	Canada, Yukon, Firth River	GenBank BOLD IDS
P. b. brevicornis	Hapl6	HQ961895.1 JSYKA236-10	n/a	Canada, Yukon, Firth River	GenBank BOLD IDS
P. b. brevicornis	Hapl7	HQ961896.1 JSYKA237-10	n/a	Canada, Yukon, Firth River	GenBank BOLD IDS
P. b. brevicornis	Hapl8	HQ961897.1 JSYKA238-10	n/a	Canada, Yukon, Firth River	GenBank BOLD IDS
P. b. brevicornis	Hapl8	HQ961898.1 JSYKA239-10	n/a	Canada, Yukon, Firth River	GenBank BOLD IDS
P. b. brevicornis	Hapl4	KJ204113.1 HMCOC006-07	n/a	Canada, Manitoba, Churchill	Woodcock, 2013

P. b. brevicornis	Hapl4	KJ203181.1 HMCOC510-09	n/a	Canada, Manitoba, Churchill	Woodcock, 2013
P. b. brevicornis	Hapl4	KJ203639.1 HMCOC627-09	n/a	Canada, Manitoba, Churchill	Woodcock, 2013
P. b. brevicornis	Hapl4	KJ203239.1 MZCHU103-07	n/a	Canada, Manitoba, Churchill	Woodcock, 2013
P. b. brevicornis	Hapl4	KJ203875.1 MZCHU107-07	n/a	Canada, Manitoba, Churchill	Woodcock, 2013
P. b. brevicornis	Hapl4	KJ204094.1 MZCHU111-07	n/a	Canada, Manitoba, Churchill	Woodcock, 2013
P. b. brevicornis	Hapl4	JN310764.1 TWCOL664-10	n/a	Canada, Manitoba, Churchill	GenBank BOLD IDS
P. b. brevicornis	Hapl4	KJ203437.1 DSCOL501-07	n/a	Canada, Manitoba, Churchill	Woodcock, 2013
P. b. brevicornis	Hapl4	KJ203343.1 HMCOC242-07	n/a	Canada, Manitoba, Churchill	Woodcock, 2013
P. b. brevicornis	Hapl4	KJ204090.1 HMCOC259-07	n/a	Canada, Manitoba, Churchill	Woodcock, 2013
P. b. brevicornis	Hapl4	KJ203213.1 HMCOC631-09	n/a	Canada, Manitoba, Churchill	Woodcock, 2013
P. b. brevicornis	Hapl4	KJ203822.1 HMCOC632-09	n/a	Canada, Manitoba, Churchill	Woodcock, 2013
P. b. brevicornis	Hapl4	KJ204238.1 HMCOC629-09	n/a	Canada, Manitoba, Churchill	GenBank BOLD IDS
P. b. brevicornis	Hapl4	HQ582357.1 TWCOL385-10	n/a	Canada, Manitoba	GenBank BOLD IDS
P. b. brevicornis	Hapl5	KJ203657.1 HMCOL237-09	n/a	Canada, Manitoba, Churchill	Woodcock, 2013
P. b. brevicornis	Hapl9	KU876036.1 UAMIC364-13	n/a	USA, Alaska, Talkeetna	Sikes, 2017
P. b. brevicornis	Hapl2	BETN4365-19	n/a	USA, Alaska, Fairbanks Borough	BOLD IDS
P. b. brevicornis	Hapl2	BETN4479-19	n/a	USA, Alaska, Fairbanks Borough	BOLD IDS
P. b. brevicornis	Hapl2	BETN4485-19	n/a	USA, Alaska, Fairbanks Borough	BOLD IDS
P. b. brevicornis	Hapl2	BETN4487-19	n/a	USA, Alaska, Fairbanks Borough	BOLD IDS
P. b. brevicornis	Hapl2	BETN4497-19	n/a	USA, Alaska, Fairbanks Borough	BOLD IDS
P. b. brevicornis	Hapl2	BETN4506-19	n/a	USA, Alaska, Fairbanks Borough	BOLD IDS
P. b. brevicornis	Hapl2	BETN647-18	n/a	USA, Alaska, Fairbanks Borough	BOLD IDS
P. b. brevicornis	Hapl2	BETN4477-19	n/a	USA, Alaska, Fairbanks Borough	BOLD IDS
P. b. brevicornis	Hapl2	BETN4488-19	n/a	USA, Alaska, Fairbanks Borough	BOLD IDS
P. b. brevicornis	Hapl2	BETN4361-19	n/a	USA, Alaska, Fairbanks Borough	BOLD IDS
P. b. brevicornis	Hapl2	BETN4480-19	n/a	USA, Alaska, Fairbanks Borough	BOLD IDS
P. b. brevicornis	Hapl2	BETN4483-19	n/a	USA, Alaska, Fairbanks Borough	BOLD IDS
P. b. brevicornis	Hapl2	BETN4484-19	n/a	USA, Alaska, Fairbanks Borough	BOLD IDS
P. b. brevicornis	Hapl2	BETN4500-19	n/a	USA, Alaska, Fairbanks Borough	BOLD IDS

P. b. brevicornis	Hapl2	BETN646-18	n/a	USA, Alaska, Fairbanks Borough	BOLD IDS
P. b. brevicornis	Hapl2	BETN7632-20	n/a	USA, Alaska, Fairbanks Borough	BOLD IDS
P. b. brevicornis	Hapl3	BETN4478-19	n/a	USA, Alaska, Fairbanks Borough	BOLD IDS
P. b. brevicornis	Hapl3	BETN4493-19	n/a	USA, Alaska, Fairbanks Borough	BOLD IDS
P. b. brevicornis	Hapl3	BETN4333-19	n/a	USA, Alaska, Fairbanks Borough	BOLD IDS
P. b. brevicornis	Hapl3	BETN4372-19	n/a	USA, Alaska, Fairbanks Borough	BOLD IDS
P. b. brevicornis	Hapl3	BETN4489-19	n/a	USA, Alaska, Fairbanks Borough	BOLD IDS
P. b. brevicornis	Hapl3	BETN4490-19	n/a	USA, Alaska, Fairbanks Borough	BOLD IDS
P. b. brevicornis	Hapl3	BETN4319-19	n/a	USA, Alaska, Fairbanks Borough	BOLD IDS
P. b. brevicornis	Hapl3	BETN4495-19	n/a	USA, Alaska, Fairbanks Borough	BOLD IDS
P. b. brevicornis	Hapl3	BETN4496-19	n/a	USA, Alaska, Fairbanks Borough	BOLD IDS
P. b. brevicornis	Hapl3	BETN4318-19	n/a	USA, Alaska, Fairbanks Borough	BOLD IDS
P. b. brevicornis	Hapl3	BETN4321-19	n/a	USA, Alaska, Fairbanks Borough	BOLD IDS
P. b. brevicornis	Hapl3	BETN4327-19	n/a	USA, Alaska, Fairbanks Borough	BOLD IDS
P. b. brevicornis	Hapl3	BETN4481-19	n/a	USA, Alaska, Fairbanks Borough	BOLD IDS
P. b. brevicornis	Hapl14	BETN7631-20	n/a	USA, Alaska, Fairbanks Borough	BOLD IDS
P. b. brevicornis	Hapl15	BETN7637-20	n/a	USA, Alaska, Fairbanks Borough	BOLD IDS
P. b. brevicornis	Hapl12	BETN7640-20	n/a	USA, Alaska, Fairbanks Borough	BOLD IDS
P. b. brevicornis	Hapl3	BETN7627-20	n/a	USA, Alaska, Southeast Fairbanks	BOLD IDS
P. b. brevicornis	Hapl2	BETN7619-20	n/a	USA, Alaska, Southeast Fairbanks	BOLD IDS
P. b. brevicornis	Hapl2	BETN4409-19	n/a	USA, Alaska, Denali Borough	BOLD IDS
P. b. brevicornis	Hapl2	BETN4428-19	n/a	USA, Alaska, Denali Borough	BOLD IDS
P. b. brevicornis	Hapl2	BETN4455-19	n/a	USA, Alaska, Denali Borough	BOLD IDS
P. b. brevicornis	Hapl2	BETN4429-19	n/a	USA, Alaska, Denali Borough	BOLD IDS
P. b. brevicornis	Hapl2	BETN4431-19	n/a	USA, Alaska, Denali Borough	BOLD IDS
P. b. brevicornis	Hapl2	BETN4470-19	n/a	USA, Alaska, Denali Borough	BOLD IDS
P. b. brevicornis	Hapl2	BETN609-18	n/a	USA, Alaska, Denali Borough	BOLD IDS
P. b. brevicornis	Hapl2	BETN4458-19	n/a	USA, Alaska, Denali Borough	BOLD IDS
P. b. brevicornis	Hapl2	BETN568-18	n/a	USA, Alaska, Denali Borough	BOLD IDS
P. b. brevicornis	Hapl2	BETN612-18	n/a	USA, Alaska, Denali Borough	BOLD IDS
P. b. brevicornis	Hapl2	BETN4420-19	n/a	USA, Alaska, Denali Borough	BOLD IDS
P. b. brevicornis	Hapl2	BETN579-18	n/a	USA, Alaska, Denali Borough	BOLD IDS
P. b. brevicornis	Hapl2	BETN636-18	n/a	USA, Alaska, Denali Borough	BOLD IDS
P. b. brevicornis	Hapl3	BETN4433-19	n/a	USA, Alaska, Denali Borough	BOLD IDS
P. b. brevicornis	Hapl11	BETN623-18	n/a	USA, Alaska, Denali Borough	BOLD IDS
P. b. brevicornis	Hapl12	BETN633-18	n/a	USA, Alaska, Denali Borough	BOLD IDS
P. b. brevicornis	Hapl10	BETN4423-19	n/a	USA, Alaska, Denali Borough	BOLD IDS
P. b. brevicornis	Hapl10	BETN4254-19	n/a	USA, Alaska, Toolik	BOLD IDS

P. b. brevicornis	Hapl10	BETN4261-19	n/a	USA, Alaska, Toolik	BOLD IDS
P. b. brevicornis	Hapl1	BETN4256-19	n/a	USA, Alaska, Toolik	BOLD IDS
P. b. brevicornis	Hapl10	MT792007	MZ366473	Russia, Chukotka, Pevek	Present study
P. b. brevicornis	Hapl18	MT792008	n/a	Russia, Chukotka, Pevek	Present study
P. b. brevicornis	Hapl18	MT792009	MZ366474	Russia, Chukotka, Pevek	Present study
P. b. brevicornis	Hapl10	MT792010	MT791964	Russia, Chukotka, Apapelgino	Present study
P. b. brevicornis	Hapl10	MT792011	n/a	Russia, Chukotka, Apapelgino	Present study
P. b. brevicornis	Hapl10	MT792012	n/a	Russia, Chukotka, Alkatvaam	Present study
P. b. brevicornis	Hapl10	MT792013	n/a	Russia, Chukotka, Alkatvaam	Present study
P. b. brevicornis	Hapl10	MT792014	n/a	Russia, Chukotka, Alkatvaam	Present study
P. b. brevicornis	Hapl21	MT792015	n/a	Russia, Chukotka, Alkatvaam	Present study
P. b. brevicornis	Hapl10	MT792016	n/a	Russia, Chukotka, Alkatvaam	Present study
P. b. brevicornis	Hapl10	MT792017	MT791965	Russia, Chukotka, Hatyrka	Present study
P. b. brevicornis	Hapl10	MT792018	MT791966	Russia, Chukotka, Hatyrka	Present study
P. b. brevicornis	Hapl10	MT792019	n/a	Russia, Chukotka, Hatyrka	Present study
P. b. brevicornis	Hapl10	MT792020	n/a	Russia, Chukotka, Hatyrka	Present study
P. b. brevicornis	Hapl10	MT792021	MT791967	Russia, Chukotka, Amguema	Present study
P. b. brevicornis	Hapl10	MT792022	MT791968	Russia, Chukotka, Amguema	Present study
P. b. brevicornis	Hapl10	MT792023	MT791969	Russia, Chukotka, Meynipil-gino	Present study
P. b. brevicornis	Hapl10	MZ351277	n/a	Russia, Chukotka, Kosa Belyaka	Present study
P. b. brevicornis	Hapl10	MZ351278	n/a	Russia, Chukotka, Kosa Belyaka	Present study
P. b. brevicornis	Hapl10	MT792024	MT791970	Russia, Wrangel Island, Som-nitelnaya Bay	Present study
P. b. brevicornis	Hapl20	MT792025	MT791971	Russia, Wrangel Island, Som-nitelnie mountains	Present study
P. b. brevicornis	Hapl20	MZ351285	n/a	Russia, Wrangel Island, Som-nitelnie mountain	Present study
P. b. brevicornis	Hapl6	MZ351282	n/a	Russia, Wrangel Island, Som-nitelnie mountains	Present study
P. b. brevicornis	Hapl10	MT792026	MT791972	Russia, Wrangel Island, Mi-neeva mountains	Present study
P. b. brevicornis	Hapl10	MT792027	n/a	Russia, Wrangel Island, Mi-neeva mountains	Present study
P. b. brevicornis	Hapl18	MT792028	MT791973	Russia, Taymyr area, Kotuy	Present study
P. b. brevicornis	Hapl18	MT792029	MT791974	Russia, Taymyr area, Kotuy	Present study
P. b. brevicornis	Hapl10	MT792030	n/a	Russia, Taymyr Peninsula, Ary-Mas	Present study
P. b. brevicornis	Hapl10	MT792031	MT791975	Russia, Taymyr Peninsula, Ary-Mas	Present study
P. b. brevicornis	Hapl18	MT792032	n/a	Russia, Taymyr Peninsula, Ary-Mas	Present study
P. b. brevicornis	Hapl18	MT792033	n/a	Russia, Taymyr Peninsula, Ary-Mas	Present study
P. b. brevicornis	Hapl18	MT792034	n/a	Russia, Taymyr Peninsula, Ary-Mas	Present study
P. b. brevicornis	Hapl18	MT792035	n/a	Russia, Taymyr area, Kotuy	Present study
P. b. brevicornis	Hapl18	MT792036	n/a	Russia, Taymyr Peninsula, Kotuy	Present study
P. b. brevicornis	Hapl18	MT792037	n/a	Russia, Taymyr Peninsula, Kotuy	Present study
P. b. brevicornis	Hapl15	MT792038	MT791976	Russia, Polar Ural, Harp	Present study
P. b. brevicornis	Hapl15	MT792039	MT791977	Russia, Polar Ural, Harp	Present study
P. b. brevicornis	Hapl15	MT792040	MT791978	Russia, Polar Ural, Harp	Present study
P. b. brevicornis	Hapl15	MT792041	MZ366462	Russia, Polar Ural, Harp	Present study
P. b. brevicornis	Hapl15	MT792042	n/a	Russia, Polar Ural, Harp	Present study

P. b. brevicornis	Hapl15	MT792043	n/a	Russia, Tazovsky Peninsula, Messoyaha	Present study
P. b. brevicornis	Hapl15	MT792044	MT791979	Russia, Tazovsky Peninsula, Messoyaha	Present study
P. b. brevicornis	Hapl15	MT792045	MT791980	Russia, Tazovsky Peninsula, Messoyaha	Present study
P. b. brevicornis	Hapl18	MT792046	n/a	Russia, Tazovsky Peninsula, Messoyaha	Present study
P. b. brevicornis	Hapl16	MT792047	MT791981	Russia, Vaigach Island, Bolvansky Nos	Present study
P. b. brevicornis	Hapl16	MT792048	MT791982	Russia, Vaigach Island, Bolvansky Nos	Present study
P. b. brevicornis	Hapl16	MZ351267	n/a	Russia, Vaigach Island, Bolvansky Nos	Present study
P. b. brevicornis	Hapl17	MZ351259	n/a	Russia, Vaigach Island, Bolvansky Nos	Present study
P. b. brevicornis	Hapl17	MZ351260	MZ366463	Russia, Vaigach Island, Bolvansky Nos	Present study
P. b. brevicornis	Hapl17	MZ351261	MZ366464	Russia, Vaigach Island, Bolvansky Nos	Present study
P. b. brevicornis	Hapl17	MZ351262	n/a	Russia, Vaigach Island, Bolvansky Nos	Present study
P. b. brevicornis	Hapl17	MZ351263	n/a	Russia, Vaigach Island, Bolvansky Nos	Present study
P. b. brevicornis	Hapl17	MZ351264	n/a	Russia, Vaigach Island, Bolvansky Nos	Present study
P. b. brevicornis	Hapl17	MZ351265	n/a	Russia, Vaigach Island, Bolvansky Nos	Present study
P. b. brevicornis	Hapl17	MZ351266	n/a	Russia, Vaigach Island, Bolvansky Nos	Present study
P. b. brevicornis	Hapl17	MZ351267	n/a	Russia, Vaigach Island, Bolvansky Nos	Present study
P. b. brevicornis	Hapl19	MT792049	MZ366465	Russia, Ygorsky Peninsula, Amderma	Present study
P. b. brevicornis	Hapl16	MT792050	MZ366466	Russia, Ygorsky Peninsula, Amderma	Present study
P. b. brevicornis	Hapl10	MT792051	n/a	Russia, Ygorsky Peninsula, Amderma	Present study
P. b. brevicornis	Hapl16	MT792052	MT791983	Russia, Ygorsky Peninsula, Amderma	Present study
P. b. brevicornis	Hapl16	MZ351269	MZ366467	Russia, Ygorsky Peninsula, Amderma	Present study
P. b. brevicornis	Hapl16	MZ351270	MZ366468	Russia, Ygorsky Peninsula, Beliy Nos	Present study
P. b. brevicornis	Hapl16	MT792053	n/a	Russia, Pinega reserve	Present study
P. b. brevicornis	Hapl16	MT792054	MT791984	Russia, Pinega reserve	Present study
P. b. brevicornis	Hapl16	MT792055	MT791985	Russia, Pinega reserve	Present study
P. b. brevicornis	Hapl22	MT792056	MT791986	Russia, Kanin Peninsula, Shoina	Present study
P. b. brevicornis	Hapl17	MT792057	MT791987	Russia, Kanin Peninsula, Shoina	Present study
P. b. brevicornis	Hapl22	MT792058	n/a	Russia, Kanin Peninsula, Shoina	Present study
P. b. brevicornis	Hapl22	MT792059	n/a	Russia, Kanin Peninsula, Shoina	Present study
P. b. brevicornis	Hapl22	MT792060	n/a	Russia, Kanin Peninsula, Shoina	Present study

P. b. brevicornis	Hapl17	MT792061	n/a	Russia, Kanin Peninsula, Shoina	Present study
P. b. brevicornis	Hapl17	MT792062	n/a	Russia, Kanin Peninsula, Shoina	Present study
P. b. brevicornis	Hapl17	MT792063	n/a	Russia, Kanin Peninsula, Shoina	Present study
P. b. brevicornis	Hapl17	MT792064	n/a	Russia, Kanin Peninsula, Shoina	Present study
P. b. brevicornis	Hapl19	MT792065	n/a	Russia, Yamal Peninsula, Sey-akha	Present study
P. b. brevicornis	Hapl23	MT792066	n/a	Kamchatka, Karaginskiy Is-land	Present study
P. b. brevicornis	Hapl23	MZ351281	n/a	Kamchatka, Karaginskiy Is-land	Present study
Out-group taxa					
Pterostichus nivalis (*R.F.Sahlberg, 1844*)	-	MT792067	MT791988	Russia, Chukotka, Apapelgino	Present study
P. nivalis	-	MT792068	MT791989	Russia, Chukotka, Hatyrka	Present study
P. nivalis	-	MT792069	MT791990	Russia, Chukotka, Kosa Belyaka	Present study

Table S2. Pterostichus brevicornis brevicornis 28S rRNA polymorphism *.

Haplotype, Code	GenBank acc. no.	173	274	276	404	423	428	429	430	447	488
Hapl 1	MT791970, MT791967, MT791968, MT791969	T	C	T	G	T	G	A	A	C	-
Hapl 2	MT791965, MT791966	·	·	·	·	C	·	·	·	·	-
Hapl 3	MT791971, MT791972, MZ366473	·	·	·	·	·	T	T	-	T	T
Hapl 4	MT791973, MT791976, MT791977, MT791978, MZ366462, MT791981, MT791979, MT791980, MZ366465, MZ366466, MZ366467 MT791983, MZ366468	A	T	-	·	·	T	T	-	T	-
Hapl 5	MT791964	A	T	-	·	·	T	T	T	·	T
Hapl 6	MT791974, MT791975	A	T	-	A	·	T	T	-	T	-
Hapl 7	MT791982, MZ366463, MZ366464, MT791984 MT791985, MT791986, MT791987	A	T	-	·	·	T	T	-	·	-
Hapl 8	MZ366474	A	T	-	·	·	T	T	T	·	-

* - Numbers in the top row refer to nucleotide positions within a condensed alignment of the sequences, letters are FASTA codes for nucleotides but with additional polymorphisms shown explicitly. Dots indicate a nucleotide matching the first sequence. Dashes indicate gaps after alignment of the sequences.

Table S3. Sampling sites and sample size: total number of pronotum (males and females) and male genitalia (aedeagus) of *Pterostichus b. brevicornis*.

Locality	Site	Sampling year	Sample size			Sequences
			Pronotum		Aedeagus	
			male	female		
Ygorsky Peninsula	Amderma settlement (Am)	2012	3	3	4	COI, 28S rRNA
Kolguev Island	Bugrino settlement (Bug)	2009	3	3	3	n/a
Kanin Peninsula	Shoina settlement (Sho)	2019	5	4	4	COI, 28S rRNA
Pinega reserve	Golubino settlement (Pin)	2019	4	3	5	COI, 28S rRNA
Vaygach Island	Bolvansky Nos (Vay)	2013	8	3	8	COI, 28S rRNA
Kola Peninsula	Khibiny mountains (Khi)	2018	3	3	-	n/a
Polar Ural	Harp settlement (PU)	2017	6	3	6	COI, 28S rRNA
Yamal Peninsula	Syoyakha settlement (Yam)	2014	5	3	5	COI
Tazovsky Peninsula	Messoyaha River (Taz)	2008	4	3	4	COI, 28S rRNA
Taymyr Peninsula	Meduza Bay (MB)	2002	-	-	2	n/a
Taymyr area	Kotuy River (T.Kr)	2010	3	3	-	COI, 28S rRNA
Taymyr Peninsula	Maksimovka River (T.Mr)	2003	3	3	-	n/a
Taymyr Peninsula	Ladannah Lake (T.Lr)	2001	8	3	8	n/a
Taymyr Peninsula	Ary-Mas (AM)	2010	3	3	-	COI, 28S rRNA
Chukotka	Pevek settlement (Pev)	2011	3	3	-	COI
Chukotka	Apapelgino settlement (Ap)	2019	3	3	-	COI, 28S rRNA
Wrangel Island	Mineeva mountains (Wr)	2015	3	3	-	COI, 28S rRNA
	**Total**		**67**	**49**	**49**	

n/a – not available.

Table S4. Contribution of significant principal components to shape variation of pronotum and aedeagus of *Pterostichus b. brevicornis*.

Components	Pronotum			Aedeagus		
	Eigenvalue	Proportion (%)	Cumulative (%)	Eigenvalue	Proportion (%)	Cumulative (%)
PC1	10.1×10^5	51.10	51.10	41.0×10^5	55.45	55.45
PC2	3.5×10^5	17.76	68.86	9.2×10^5	12.40	67.85
PC3	2.3×10^5	11.66	80.52	8.1×10^5	11.03	78.87
PC4	1.2×10^5	5.86	86.38	4.2×10^5	5.70	84.57
PC5	0.8×10^5	3.85	90.22	2.5×10^5	3.40	87.98

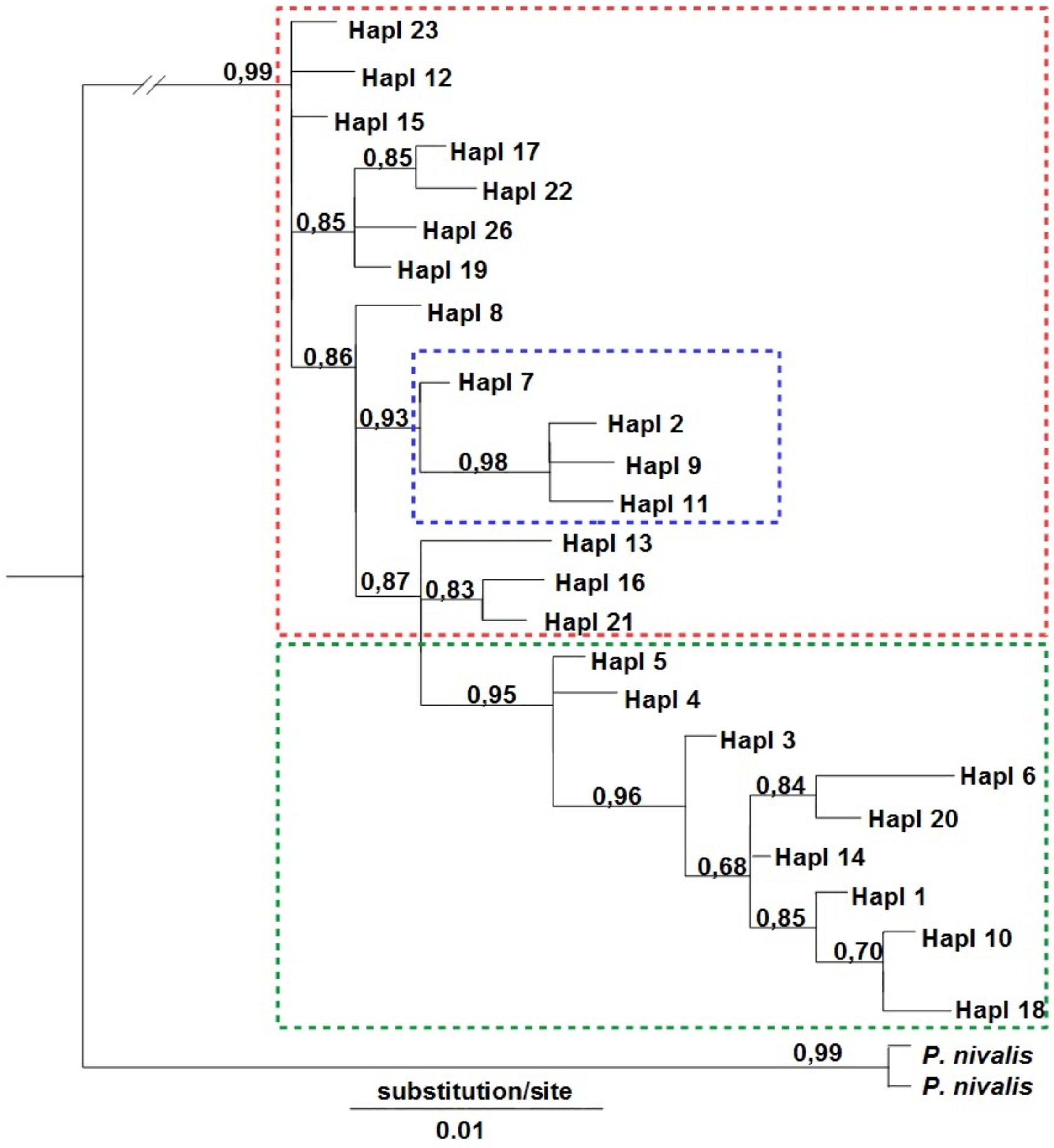

Figure S1. Phylogenetic tree of *Pterostichus brevicornis brevicornis* recovered from Bayesian inference analysis based on the COI sequence dataset. Haplotype codes are indicated in Table S1. The dashed rectangles corresponded to subclades of the majority-rule consensus phylogenetic tree (see Figure 2).Numbers near branches indicate the Bayesian posterior probability (BPP).
